# Efficacy of Mat Pilates on the resting blood pressure and health related quality of life in individuals with systemic hypertension versus standard care: study protocol for a single centered single blinded randomized controlled trial

**DOI:** 10.12688/f1000research.146980.1

**Published:** 2024-07-26

**Authors:** Nivedita S. Prabhu, G. Arun Maiya, Vaishali K, Shivashankara KN

**Affiliations:** 1 Department of Physiotherapy, Manipal College of Health Professions, Manipal Academy of Higher Education, Manipal, Karnataka, 576104, India; 2 Department of Physiotherapy, Manipal College of Health Professions, Manipal Academy of Higher Education, Manipal, Karnataka, 576104, India; 3 Department of Physiotherapy, Manipal College of Health Professions, Manipal Academy of Higher Education, Manipal, Karnataka, 576104, India; 4 Department of Medicine, Kasturba Medical College, Manipal Academy of Higher Education, Manipal, Karnataka, 576104, India

**Keywords:** exercise, blood pressure, quality of life, Pilates, movement therapy

## Abstract

**Background:**

Systemic hypertension is a global non-communicable disease that creates an essential need for alternate forms of lifestyle modifications, including exercise, to lower elevated blood pressure. Mat Pilates, a feasible form of mind-body coordinated exercise, may provide benefits with limited resources.

**Objective:**

This randomized controlled trial aimed to assess the efficacy of Mat Pilates on resting blood pressure and health-related quality of life in individuals with systemic hypertension.

**Methods:**

A two-arm, single-blinded, block randomized controlled trial will be recruited with120 participants into control and experimental groups (1:1 ratio). Mat Pilates will be administered to the experimental group and standard care to the control group for 12 weeks. Resting and central blood pressure, health-related quality of life, and echocardiographic parameters will be measured before and after the trial. We hypothesized that Pilates may not be beneficial in lowering resting blood pressure in hypertensive patients. A repeated-measures analysis of variance (ANOVA) will test the within- and between-group effects of Mat Pilates on various outcomes.

**Ethics and Dissemination:**

The trial was approved by the Institutional Research Committee, Manipal College of Health Professions, Manipal Academy of Higher Education, Kasturba Medical College, and Kasturba Hospital Institutional Ethics Committee, Manipal. Written informed consent will be obtained from all the participants. All stakeholders and committees will communicate key findings regarding the implementation of mind-body association exercises as a measure of lifestyle modification in individuals with systemic hypertension.

**Trial registration:**

Clinical Trials Registry of India: CTRI/2021/07/035002. Registered on July 20, 2021, http://ctri.nic.in.

**Abbreviations**
ANOVA: Analysis of Variance
CVD: Cardiovascular Disease
HrQOL: Health related Quality of Life
JNC 7: Seventh Joint National Committee Report
LVEF: Left Ventricular Ejection Fraction
WHO: World Health Organization

## Article summary

Strengths and Limitations of this study

• This single-blinded, randomized controlled trial will enable awareness of a feasible, cost-effective form of Mat Pilates exercise for participants diagnosed with hypertension that can be easily implemented in low-resource settings.
• As a mind-body association exercise, individuals with systemic hypertension would benefit from relaxed breathing maneuvers, which will in turn aid in the relaxation of various muscle groups and peripheral vasculature.
• As this is a single-center trial, this study may not be accessible to participants outside the study setting. In addition, Mat Pilates needs supervision for a minimum of 2 weeks to ensure the correct learning of exercise patterns.

## Introduction

Systemic arterial hypertension is a significant contributor to the non-communicable disease spectrum. The World Health Organization (WHO) reports that systemic hypertension accounts for almost 45% and 51% of mortality from heart disease and stroke worldwide, respectively. ^1^ The Global Action Plan for the control and prevention of non-communicable diseases (NCDs) aims to reduce the prevalence of systemic hypertension by the year 2025. ^2^ The South Asian subcontinent is undergoing extensive economic growth, leading to inimical urban lifestyle changes. A mortality rate of 1.5 million deaths per year has been reported due to cardiovascular disease (CVD) among Indians, and by 2020, CVD will be the leading cause of mortality and morbidity. ^3^^,^^4^ Therapeutic modes adopted for systemic hypertension include antihypertensive drug therapy, lifestyle modifications such as diet, and regular physical activity or exercise. Dietary Approaches to Stop Hypertension (DASH) and regular aerobic exercise for 30 minutes per day are mandated determinants to lower elevated blood pressure. ^5^^–^^7^ Meta-analyses of various randomized controlled trials report dual benefits of aerobic and resistance exercise in lowering blood pressure. ^8^^,^^9^ However, these studies sparsely include alternate approaches of mind-body exercises such as Pilates, Tai Chi, Yoga, etc.

### Application of Pilates exercise

The incorporation of the Pilates method of exercise differs from routine structured physical exercise protocols, as it is based on a mind-body association. A thought process used positively to execute a particular movement with specific breathing patterns, generating kinesthetic awareness aids in reducing mental stress, anxiety, enhancing sleep, and a sense of well-being. Changes in body movement as a whole with concentration, synchronized breathing, and precise control of the body part to be moved with precision in a rhythmic manner creates an environment of the movement pattern and ability to identify the muscle being recruited to perform the desired movement. ^10^^–^^14^ Pilates can be performed either on the mat or using Pilates equipment using one’s body weight. ^15^^,^^16^ It has been effectively reviewed on various health-related parameters such as body composition, ^17^ stress urinary incontinence, ^18^ and breast cancer survivors’ health-related quality of life, ^19^ falls, and physical fitness in the elderly. ^20^^–^^22^

### Merits of Mat Pilates

Following the principles of control, breathing, concentration, flow of movement, centering, and Pilates have often been used to improve physical function and muscle conditioning. The benefits of Mat Pilates being a feasible, novel, and recreational mode of exercise implementing cost-effective resources of mats has been reported in different populations such as multiple sclerosis, Parkinson’s disease, older adults, and low back pain. ^23^^–^^26^ Energy expenditure (EE), blood lactate levels, oxygen uptake (VO2 uptake), and low cardiovascular stress (including heart rate and blood pressure) have been observed with the implementation of Mat Pilates when compared to the Reformer Pilates method (which utilizes larger equipment) in healthy young female adults. ^27^ Although the above studies report beneficial effects of Mat Pilates on a spectrum of disease conditions, there is a scarcity of studies reporting the effects of mat Pilates on variations in peripheral and central blood pressure parameters in individuals with systemic hypertension. In addition, structural and hemodynamic changes within the cardiac chambers have rarely been reported in relation to this exercise method in hypertensive individuals.

### Objectives

The primary and secondary objectives of this trial were to study the effects of mat Pilates on.

*Primary outcomes*:

1. Resting blood pressure (systolic and diastolic blood pressure) (mm of Hg)
2. Central systolic blood pressure and pulse pressure (mm of Hg)
3. Mean arterial pressure (MAP) (mm of Hg)

*Secondary outcomes*:

4. Health related quality of life (HrQoL)
5. Two-dimensional (2D) echocardiographic parameters included the left ventricular ejection fraction (LVEF) and end-diastolic volume.

*Hypotheses*:

Null hypothesis: This trial hypothesizes that mat Pilates will not have a beneficial reduction in arterial blood pressure and will not improve health-related quality of life in participants with systemic hypertension.

Alternate hypothesis: Mat Pilates has a positive effect on reducing resting blood pressure, including central pulse pressure and central systolic pressure. In addition, the application of mat Pilates will improve health-related quality of life in individuals with systemic hypertension.

### Study design

Considering the fewer studies reported, this trial will be a two-arm, single-blinded, prospective randomized controlled trial (RCT) aimed at evaluating the efficacy of a 12 weeks, mat Pilates program on changes in resting and central blood pressures (central pulse pressure and central systolic pressure) in a total of 120 participants.

### Study setting

Participants will be screened in the outpatient departments of General Medicine and Cardiology, Kasturba Hospital, Manipal.

Mat Pilates exercises will be administered and supervised in the outpatient department of the Physiotherapy and Health Performance Laboratory, Kasturba Hospital Manipal.

### Participants

Participants aged between 30 and 60 years diagnosed with systemic arterial hypertension with optimal blood pressure control on regular antihypertensive therapy will be recruited for this clinical trial.

### Eligibility criteria

*Inclusion criteria*:

1. Stage 1 (systolic blood pressure > 130–139 mmHg or diastolic blood pressure > 80–89 mmHg) and Stage 2 (systolic blood pressure >140–159 mmHg or diastolic blood pressure > 90–99 mmHg) hypertensive individuals.

*Exclusion criteria*:

1. Stage 2 hypertension: systolic blood pressure >160 mmHg and diastolic blood pressure >100 mmHg.
2. Accelerated hypertension/Hypertensive crisis.
3. Any discontinuation or change in the antihypertensive drug regime during the study period.
4. Diagnosed cases of acute myocardial infarction, valvular heart disease (aortic, mitral, tricuspid, pulmonic), arterial aneurysms with impending cardiac arrhythmias (on external or internal pacing devices, implanted cardioverter defibrillator (ICD).
5. Pregnancy and lactating hypertensive women.
6. Individuals with cancer on previous or current chemotherapy, radiation therapy.
7. Acute or chronic renal insufficiency on peritoneal or hemodialysis.
8. Individuals with neurological disease causing impaired cognition and incoordination of movements.
9. Musculoskeletal trauma with disability restricting movement patterns.
10. Individuals with vertigo
11. Individuals unable to comprehend instructions for the exercise program.

## Methods

### Ethics approval and consent to participate

The study protocol was approved by the Kasturba Medical College and Kasturba Hospital Institutional Ethics Committee, Kasturba Hospital, Manipal (an associate hospital of the Manipal Academy of Higher Education, Manipal, Karnataka, India) (registration number: ECR/146/Inst/KA/2013/RR-19) to recruit participants (IEC: 596/2019. Date of approval September 11, 2019). The trial has been registered under the Clinical Trials Registry of India (CTRI/2021/07/035002. Registered on July 20, 2021, http://ctri.nic.in.) This clinical trial adheres to the guidelines and ethical principles for medical research of the Declaration of Helsinki involving human participants.

Any protocol-related amendments during the trial tenure will be communicated to the Institutional Research Committee, Institutional Ethics Committee, Directorate of Research, Manipal Academy of Higher Education, and Clinical Trial Registry of India. Written informed consent will be obtained from all the participants. Stakeholders and committees will receive key findings regarding the implementation of mind-body association exercises as a measure of lifestyle modification in individuals with systemic hypertension. Post-trial ancillary care will be provided to participants in the control group after completion of the clinical trial period if they wish to undergo intervention group exercises.

### Sample size determination

The calculated sample size was based on the primary outcome of resting BP considering a level of significance (α) of 0.05, with a power of 80%. With an anticipated standardized difference of four standard deviations of the primary outcome, the estimated minimal clinically important difference (MCID) at 2 mmHg after a period of 12 weeks (at baseline and after 12th week) in both the control and experimental groups, a sample of 44 participants in each group was calculated. Considering an attrition of 20%, the sample size was calculated to be 55 participants and rounded up to 60 participants per group owing to block randomization.

### Sampling method, randomization and allocation

A random number sequence will be generated using computerized random number generator. Participants will be randomized to the control and intervention groups with 1:1 allocation and a block size of 6 consisting of ten participants in each block per group. The allocation sequence will be generated using computer-generated randomized allocation software. Using the sequentially numbered opaque envelope (SNOSE) method, allocation concealment is performed. Of the 120 participants, 60 will be allocated to the control or experimental group.

### Blinding

This RCT will be a single-blind study. Random allocation to both groups will be performed by a health professional who is not a member of the investigator team. At the completion of twelve weeks, a blinded outcome assessor will assess set outcomes of the respective groups.

### Procedure

Prior permission was sought from the copyright publishers for the administration of the World Health Organization (WHO) BREF Health-Related Quality of Life Questionnaire in two languages. Participants will be screened for eligibility based on their diagnosis and stage of systemic hypertension. Eligible participants will then be familiarized with the study using the participant information sheet, and willing participants will be recruited after obtaining their written informed consent. Preliminary screening will be implemented and written informed consent will be obtained at the outpatient clinic of the Department of Medicine, Kasturba Hospital, Manipal, Karnataka, India.

Baseline assessment of three resting blood pressure measurements in supine lying position with a rest period of one minute in between measurements will be recorded at the same time of the day (10 am). Participants will be instructed to abstain from consumption of alcohol, caffeine, or any caffeine-containing beverage 10 hours prior to the exercise testing session. The values of central systolic blood pressure, central pulse pressure, and mean arterial pressure will be measured by the principal investigator. Left ventricular ejection fraction (LVEF) and left ventricular end-diastolic volume will be measured by a professional expert trained in echocardiography. Including demographic data, the baseline scores of the WHO BREF HRQOL, anthropometric measurements, and exercise stress testing will be measured. This time point will be denoted as T0. Baseline assessment of eligible participants will be performed at the Health Performance Laboratory, Department of Physiotherapy, Clinical Teaching Center, and echocardiographic measurements will be recorded at the Department of Cardiology, Kasturba Hospital, Manipal, India. The participants will then be sequentially block randomized into two groups by a blinded investigator.

Participants in the experimental group will undergo a two-week supervised Mat Pilates program, after which exercises will be performed at home for 10 weeks. An exercise instruction manual for the mat Pilates program will be provided to every participant at the end of 2 weeks. A weekly telephonic, supervised monitoring call will be implemented to address and record any issues related to compliance, adherence, or adverse events. In addition, they will be advised to follow standard care with the recommended salt intake of 2.4 g) or sodium chloride (6 g) per day and regular physical activity of 3-5 days per week. ^28^

The control group will receive standard care and a prescribed antihypertensive drug regimen. After 12 weeks, the outcomes will be reassessed and recorded (T1) by a blinded investigator. Adherence will be set at 75% of the total number of sessions (27 of 36 sessions). A flowchart of the procedure is shown in Figure 1. The schedule of participant enrolment, interventions, and assessments has been provided ( Figure 2) according to the Standard Protocol Items: Recommendations for Intervention Trials (SPIRIT) guidelines. ^29^

**Figure 1.**
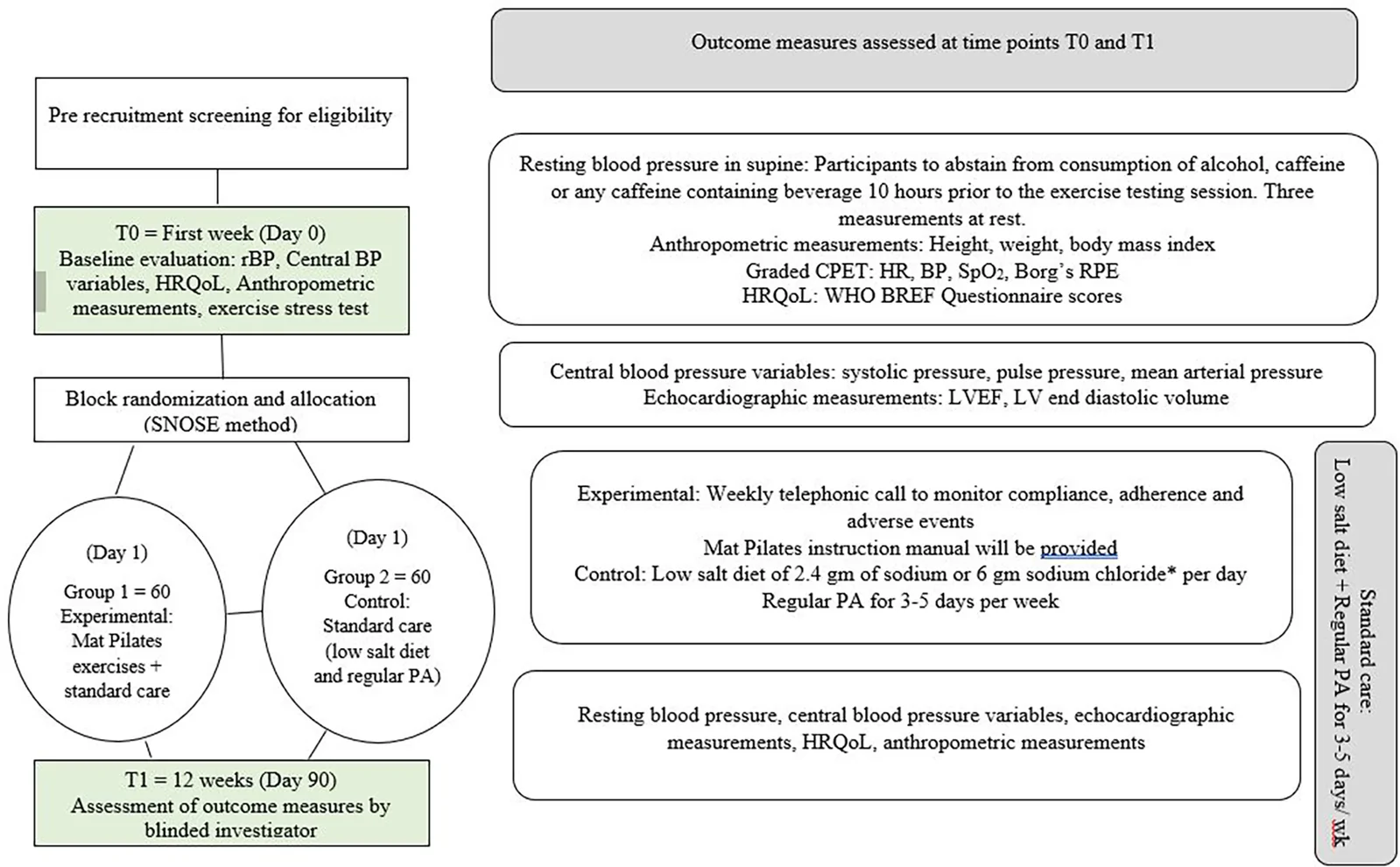
Randomised controlled trial protocol. T0, T1 Time points at baseline (T0) and after 12 weeks (T1); BP, blood pressure; CPET, cardiopulmonary exercise stress test; HrQoL, health related quality of life; LV, left ventricle; LVEF, left ventricular ejection fraction; RPE, rating of perceived exertion; SNOSE, sequentially numbered opaque sealed envelope; WHOQOL-BREF, World Health Organization Quality of Life Brief version.

**Figure 2.**
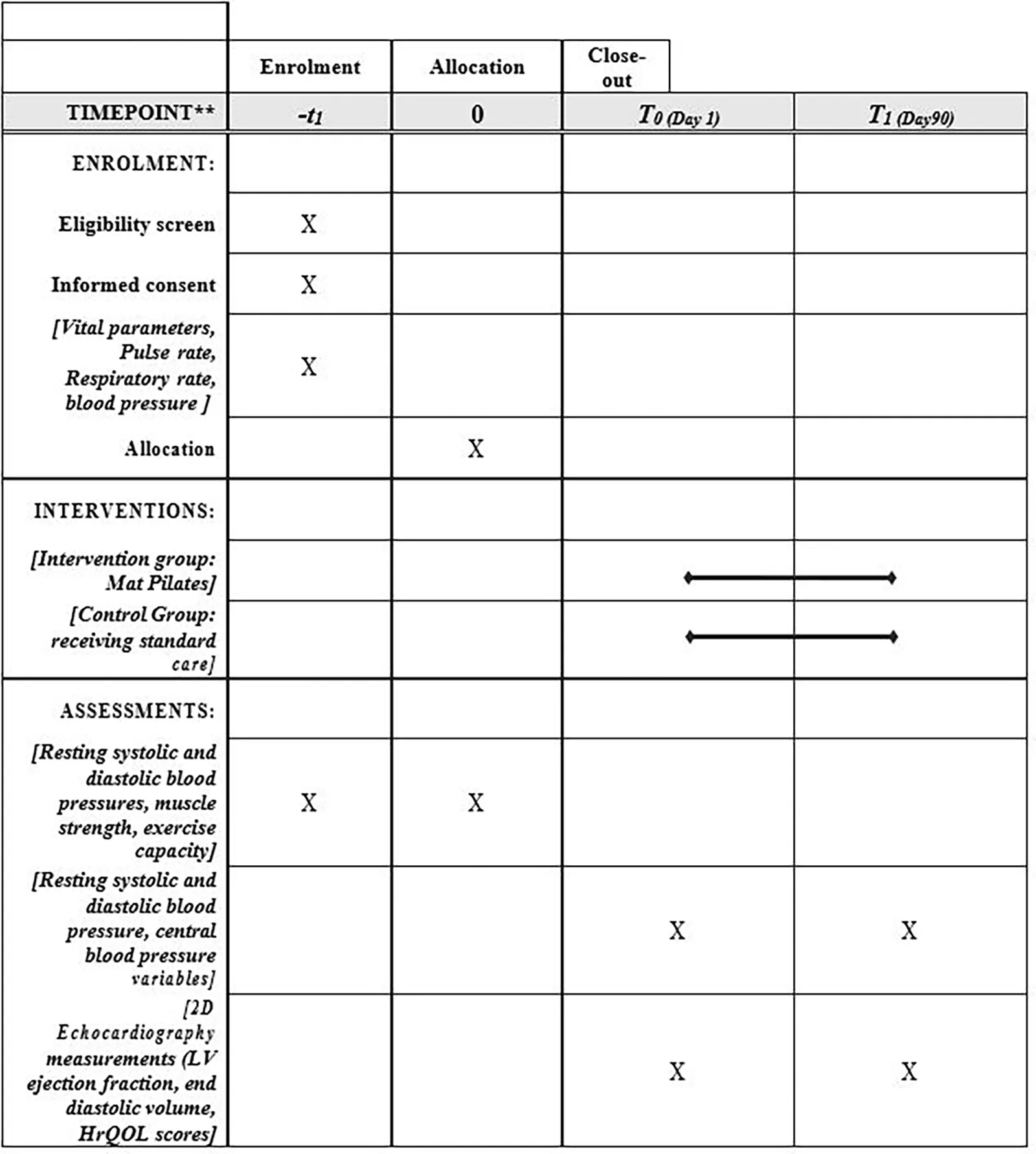
Clinical trial schedule of enrolment, interventions and assessments as per the SPIRIT guidelines. ^32^ T0, T1, Time points at baseline (T0) and after 12 weeks (90 days) (T1); 2D Echocardiography, Two dimensional echocardiography; LV, Left ventricular; HRQoL, health related quality of life.

### Intervention: Mat Pilates exercise program

The Mat Pilates exercise program will consist of 11 different exercises with a warm-up of 15 min and cool down for 10 min. Three sessions per week of 60 minutes each will be implemented ( Table 1). The exercises will be familiarized with, taught, and monitored by a certified mat Pilates instructor.

**Table 1.** Details of Mat Pilates exercises.

	Description/Instructions
Warm Up	Muscle groups stretches: (10-15 minutes) Upper Quadrant: Pectoralis major/Minor muscles, Biceps brachii, Triceps brachii, Muscles of the neck Trunk: Abdominals (Rectus abdominus, Internal and External Obliques), Back extensors (Erector Spinae, Latissimus Dorsi, Upper, Middle, Lower fibers of Trapezius) Lower quadrant: Hip flexors (Iliacus and Psoas Major), Quadriceps, Hamstrings, Tensor Fasciae Lata, Gastrocnemius, Soleus complex, Tibialis Anterior
**Mat Pilates Exercises**	
Ab Prep	Lie on your back with hip and knees bent, feet placed on the mat maintaining arms by the side. Slow breaths to be taken with elongating the spine on the mat. With exhalation, lift head, neck and shoulders with arms facing forward towards the knees. Avoid any breath hold during this process. Repeat.
Breast stroke Preps/Breast Stroke	Lie on your belly with face facing downwards, arms straight by the side. Breathe in. With exhalation, slowly lift your belly button inwards, lift head, neck and shoulders off the floor. Repeat inhalation as exhale lower back down.
Shell stretch	Kneel on the mat and rest the buttocks on the heels. Embrace your belly button to your spine. Let your spine round forward as your upper body folds toward the floor. Extend arms forwards and out straight and place your palms on the floor. With straight elbows, this is the Shell stretch. If you bring your arms by your side, you are in a resting Shell stretch. Inhale through your nose and exhale through the mouth.
One Leg Circle	Draw one leg in towards the chest and straighten it toward the ceiling. Inhale and take the leg up and over the body across the opposite shoulder. As you exhale, circle the leg down towards the center in a circling motion.
Spine Twist	In sitting with legs straightened on the mat, imagine a line running up through spine of your body. Exhale, turn your upper body and head on that central axis, getting taller as you twist. Inhale and return to the starting position. Exhale and take the twist to the other side.
Single Leg Stretch	Lie on your back with your knees bent and legs parallel to the floor in the table top position. Pull abdomen in as you exhale, taking your belly button down toward your spine as you slowly curl your head and shoulders up to the tips of the shoulder blades. Avoid any breath hold maneuvers.
Obliques (Planks – High and low)	Start with hip and knees bent with feet rested on mat. Exhale to lift and rotate your shoulder to the opposite outer knee. Focus on shifting one side of your ribcage to the opposite hip.
Heel squeeze Prone	Lie on your abdomen with face rested on both forearms. Bend both knees keeping them at the width of your shoulders to engage the muscles of the buttocks. As you squeeze the heels together imagine the thigh bones being pulled away and then up as to use your buttock muscles.
Side Leg Lift series	Lying on your side, ensure the hips, knees and ankles are aligned. Move your legs slightly in front, rest your head on your hand stretched to ensure good balance. Pull your abdominal muscles in. Inhale, allowing your body to elongate as the breath moves down the full length of your spine. With exhalation, engage your abdominals and lift your leg a few inches off the mat. Inhale, lower your legs back down to the mat.
Saw (side lying)	Sit up straight on your hip bones with legs straightened and extended forward. Stretch out both arms to your sides with arms facing downwards. As you inhale, rotate your spine and turn towards right/left activating the abdominals. As you exhale, rotate back to the starting position.
Single leg extension	Lie on your back with hips and knee bent at 90 degrees (tabletop position) with hands by the side. Exhale, bring up head and shoulders, straighten one leg with inhalation take the other leg towards the chest. Repeat with the other leg.
Toe taps	Lie on your back with hip and knees bent at 90 degrees (tabletop position) with spine in neutral position. As you exhale, lower one leg touching the toes to the mat. Inhale and bring it back to the tabletop position. Alternate and repeat with the other leg.
Cool down	Slow walking followed by slow sustained muscle stretches, slow sustained, deep breaths: (10 minutes)

### Adverse events

Adverse events will be recorded and reported every week during telephonic monitoring. Participants will be questioned regarding any major or minor events that may have occurred during the course of their exercise program tenure. The reporting of events, if any, will be implemented by the Institutional Ethics committee for further proceedings.

### Materials and equipment

The measuring equipment used will be standardized and calibrated periodically every 3 months. The Diamond ^®^ Aneroid (BPDL-250 Dial Deluxe) pressure monitor with field calibration feature will be used to measure the resting blood pressure. Mats of six feet by four feet with one and a half inch thickness will be used to perform mat Pilates exercises. The Microlife ^®^ WatchBP Office Central, which is an oscillometric calibrated blood pressure device, will measure central blood pressure parameters non-invasively using an arm cuff. Additionally, this device could detect any ongoing atrial fibrillation in the participant. The balance protocol for exercise stress testing will be performed on a Stayfit ^®^ testing treadmill.

### Outcomes

*Primary*

Resting blood pressure values (systolic/diastolic) in mmHg will be assessed at T0 and T1. Central blood pressure variables (systolic/diastolic/pulse) will be assessed non-invasively pre- and post-intervention, indicating target organ perfusion and perfusion pressure in the central organs (cerebrum, myocardium, kidneys, and liver). Changes observed in central blood pressure with regular exercise would aid in identifying any progression or reduction in target organ health in systemic hypertension.

*Secondary*

Health-related quality of life (HrQOL) in the mental, physical, and psychosocial domains will be assessed at T0 and T1, indicating any variation in the function of daily living in individuals with systemic hypertension. Two-dimensional echocardiographic findings (LVEF, left ventricular end diastolic volume) as secondary outcomes will aid in identifying any structural, ventricular wall stress-related changes in the myocardial wall before and after 12 weeks of the mat Pilates program versus standard care.

### Statistical analysis

Data will be statistically analyzed using the Jamovi software. Descriptive analysis will be used to interpret demographics, intention-to-treat analysis will be used for missing data, and subgroup analysis based on age will be used to interpret age-wise changes in blood pressure. The Kolmogorov–Smirnov test will be used as a test of normality, and repeated-measures analysis of variance (ANOVA) will be used to analyze the within-group and between-group differences and pre- and post-differences at T0 and T1. Analysis of covariance (ANCOVA) will be used to analyze the contribution of covariates in the study. All analyses of recorded data will comply with the standards of data analysis for randomized controlled trials that include the analysis of missing data due to attrition.

## Discussion

This randomized controlled trial study protocol highlights the effect of a 12 weeks mat Pilates exercise program on various blood pressure variables. This is one of the first studies to report the efficacy of Pilates exercises and changes in central blood pressures. The strengths of this study include changes in central and peripheral blood pressures, which may reduce cardiovascular stress induced in individuals with systemic hypertension. Changes in left ventricular volumes and myocardial wall thickness could be determined, thereby aiding in a cost-effective method to exercise in hypertensive individuals. Increased central blood pressure causes target organ hypoperfusion, leading to early end organ failure.

As this trial is a prospective, single-blinded study, the probability of selection bias would be reduced. An added advantage of a home-based program after a two week supervised mat Pilates exercise program would support participants in comprehending the need to exercise better, making this program cost-effective, feasible, and reduce frequent center-based visits. Although home-based, lack of supervision during the exercise regime may be a limitation of this trial. Secondary limitations would include communication between participants in both groups, which could alter exercise behavior, and alternate options for training at other exercise centers. However, if this RCT proves to be effective, it will be the one of the first studies to assess the efficacy of Mat Pilates on central blood pressure variables and ventricular remodeling related to probable changes in ventricular mass-to-volume ratios.

## Ethics approval and consent to participate

The study protocol was approved by the Kasturba Medical College and Kasturba Hospital Institutional Ethics Committee, Kasturba Hospital, Manipal (an associate hospital of the Manipal Academy of Higher Education, Manipal, Karnataka, India) (registration number: ECR/146/Inst/KA/2013/RR-19) to recruit participants (IEC: 596/2019. Date of approval September 11, 2019). The trial has been registered under the Clinical Trials Registry of India (CTRI/2021/07/035002. Registered on July 20, 2021, http://ctri.nic.in.) This clinical trial adheres to the guidelines and ethical principles for medical research of the Declaration of Helsinki involving human participants.

Any protocol-related amendments during the trial tenure will be communicated to the Institutional Research Committee, Institutional Ethics Committee, Directorate of Research, Manipal Academy of Higher Education, and Clinical Trial Registry of India. Written informed consent will be obtained from all the participants. Stakeholders and committees will receive key findings regarding the implementation of mind-body association exercises as a measure of lifestyle modification in individuals with systemic hypertension. Post-trial ancillary care will be provided to participants in the control group after completion of the clinical trial period if they wish to undergo intervention group exercises.

## Consent for publication

Not applicable.

## Patient or public involvement

Patients or the public were not involved in designing, conducting, or disseminating methods in our research.

## Author contributions

NSP conceptualized the study concept, objectives, design, performed a preliminary and ongoing literature review, and drafted the manuscript. SKN is a clinical expert in the field of systemic hypertension who performed a literature review and scrutinized the final draft of the manuscript. GAM and VK aided in the study conception, scrutinizing, and preliminary drafting of the manuscript.

## Authors’ information

NSP is currently an Assistant Professor pursuing her PhD in the Department of Physiotherapy, Manipal College of Health Professions, Manipal Academy of Higher Education with more than 12 years of teaching and research experience in the field of cardiac and pulmonary sciences. SKN is currently Professor in the Department of Medicine, Kasturba Medical College, Manipal Academy of Higher Education and has over 25 years of research and clinical expertise in the area of non-communicable diseases such as hypertension and diabetes mellitus. AGM is currently Professor and Dean, Department of Physiotherapy, Manipal College of Health Professions, Manipal Academy of Higher Education with more than 30 years of research experience in the field of diabetes mellitus and rehabilitation. VK is currently Professor, Department of Physiotherapy, Manipal College of Health Professions, Manipal Academy of Higher Education, with over 25 years of experience in the areas of geriatrics and pulmonary rehabilitation.

## Data Availability

No data were associated with this article. The World Health Organization Quality of Life BREF Questionnaire (English Version) ^30^ will be used for this study for participants fluent in English and Kannada version will be used for participants fluent in Kannada, a regional language from the state of Karnataka. ^31^ Dataverse: SPIRIT Guidelines checklist: Checklist for Efficacy of Mat Pilates on the resting blood pressure and health related quality of life in individuals with systemic hypertension versus standard care: study protocol for a single centered single blinded randomized controlled trial, DOI: https://doi.org/10.7910/DVN/GLMROL.

## References

[1] *Causes of Death 2008 [online database].* Geneva: World Health Organization. Reference Source Reference Source

[2] Global action plan for the prevention and control of noncommunicable diseases 2013-2020. Reference Source

[3] Gaziano T Reddy KS Paccaud F : Cardiovascular disease. Jamison DT Mosley WH , editors. *Disease Control Priorities in the Developing World.* Oxford: Oxford University Press;2006; p.645–62.

[4] World Health Organization: *The World Health Report 2002: Reducing Risk, Promoting Healthy Life.* Geneva, Switzerland: World Health Organization;2002.

[5] Sacks FM Svetkey LP Vollmer WM : Effects on blood pressure of reduced dietary sodium and the Dietary Approaches to Stop Hypertension (DASH) diet. DASH-Sodium Collaborative Research Group. *N. Engl. J. Med.* 2001;344:3–10. 10.1056/NEJM200101043440101 11136953

[6] Kelley GA Kelley KS : Progressive resistance exercise and resting blood pressure: A meta-analysis of randomized controlled trials. *Hypertension.* 2000;35:838–843. 10.1161/01.HYP.35.3.838 10720604

[7] Whelton SP Chin A Xin X : Effect of aerobic exercise on blood pressure: A meta-analysis of randomized, controlled trials. *Ann. Intern. Med.* 2002;136:493–503. 10.7326/0003-4819-136-7-200204020-00006 11926784

[8] Reddy KS Prabhakaran D Chaturvedi V : Methods for establishing a surveillance system for cardiovascular diseases in Indian industrial populations. *Bull. World Health Organ.* 2006;84:461–469. 16799730 10.2471/BLT.05.027037 PMC2627369

[9] Hypertension Study Group: Prevalence, awareness, treatment and control of hypertension among the elderly in Bangladesh and India: A multicenter study. *Bull. World Health Organ.* 2001;79:490–500. PMC2566443 11436469

[10] Lange C Unnithan VB Larkam E : Maximizing the benefits of Pilates-inspired exercise for learning functional motor skills. *J. Bodyw. Mov. Ther.* 2000;4:99–108. 10.1054/jbmt.1999.0161

[11] Pilates J Miller W : Return to life through contrology. Incline Village: Presentation Dynamics. 1945.

[12] Pilates JH : Your health. Incline Village: Presentation Dynamics. 1934.

[13] Latey P : The Pilates method: history and philosophy. *J. Bodyw. Mov. Ther.* 2001;5:275–282. 10.1054/jbmt.2001.0237

[14] Lessen D The PMA : *Pilates certification exam study guide.* Miami: Pilates Method Alliance;2014.

[15] Anderson B : Fitting Pilates into a rehabilitation practice. *Rehab Manag.* 2010;23(24):26–27. 20527630

[16] Kloubec J : Pilates: how does it work and who needs it? *Muscles Ligaments Tendons J.* 2011;1:61–66. 23738249 PMC3666467

[17] Aladro-Gonzalvo AR Machado-Díaz M Moncada-Jiménez J : The effect of Pilates exercises on body composition: a systematic review. *J. Bodyw. Mov. Ther.* 2012;16:109–114. 22196436 10.1016/j.jbmt.2011.06.001

[18] Bo K Herbert RD : There is not yet strong evidence that exercise regimens other than pelvic floor muscle training can reduce stress urinary incontinence in women: a systematic review. *J. Physiother.* 2013;59:159–168. 23896331 10.1016/S1836-9553(13)70180-2

[19] Stan DL Collins NM Olsen MM : The evolution of mindfulness-based physical interventions in breast cancer survivors. *Evid Based Complementary Altern Med.* 2012;2012:1–15. 10.1155/2012/758641 PMC3446749 22997532

[20] Granacher U Gollhofer A Hortobagyi T : The importance of trunk muscle strength for balance, functional performance, and fall prevention in seniors: a systematic review. *Sports Med.* 2013;43:627–641. 23568373 10.1007/s40279-013-0041-1

[21] Cancela JM Oliveira IM de Rodriguez FG : Effects of Pilates in physical fitness on older adults. A systematic review. *Eur. Rev. Aging Phys. Act.* 2014;11:81–94. 10.1007/s11556-014-0143-2

[22] Barker AL Bird ML Talevski J : Effect of Pilates exercise for improving balance in older adults: a systematic review with meta-analysis. *Arch. Phys. Med. Rehabil.* 2015;96:715–723. 25511371 10.1016/j.apmr.2014.11.021

[23] Bulguroglu I Guclu-Gunduz A Yazici G : The effects of Mat Pilates and Reformer Pilates in patients with Multiple Sclerosis: A randomized controlled study. *NeuroRehabilitation.* 2017 Jan 1;41(2):413–422. 28946576 10.3233/NRE-162121

[24] Suárez-Iglesias D Miller KJ Seijo-Martínez M : Benefits of Pilates in Parkinson’s disease: a systematic review and meta-analysis. *Medicina.* 2019 Aug;55(8):476. 31412676 10.3390/medicina55080476 PMC6723274

[25] Bertoli J Biduski GM Rocha FC de la : Six weeks of Mat Pilates training are enough to improve functional capacity in elderly women. *J. Bodyw. Mov. Ther.* 2017 Oct 1;21(4):1003–1008. 29037615 10.1016/j.jbmt.2016.12.001

[26] Da Luz MA Jr Costa LO Fuhro FF : Effectiveness of mat Pilates or equipment-based Pilates exercises in patients with chronic nonspecific low back pain: a randomized controlled trial. *Phys. Ther.* 2014 May 1;94(5):623–631. 24435105 10.2522/ptj.20130277

[27] Souza AL de Silva AI da Mochizuki L : What is the exercise intensity of Pilates? An analysis of the energy expenditure, blood lactate, and intensity of apparatus and mat Pilates sessions. *J. Bodyw. Mov. Ther.* 2021 Apr 1;26:36–42. 10.1016/j.jbmt.2020.12.007 33992270

[28] Hypertension Study Group: Prevalence, awareness, treatment and control of hypertension among the elderly in Bangladesh and India: A multicenter study. *Bull. World Health Organ.* 2001;79:490–500. PMC2566443 11436469

[29] Chan A-W Tetzlaff JM Gøtzsche PC : SPIRIT 2013 Explanation and Elaboration: Guidance for protocols of clinical trials. *BMJ.* 2013;346:e7586. 23303884 10.1136/bmj.e7586 PMC3541470

[30] Chan A-W Tetzlaff JM Altman DG : SPIRIT 2013 Statement: Defining Standard Protocol Items for Clinical Trials. *Ann. Intern. Med.* 2013;158:200–207. 23295957 10.7326/0003-4819-158-3-201302050-00583 PMC5114123

[31] Prabhu N : SPIRIT Guidelines checklist. *Harvard Dataverse.* 2024;V1. 10.7910/DVN/GLMROL

[32] WHOQOL: Measuring Quality of Life, “WHOQOL-BREF”. Reference Source

