# Supplementary material for: Efficacy of Mat Pilates on the resting blood pressure and health related quality of life in individuals with systemic hypertension versus standard care: study protocol for a single centered single blinded randomized controlled trial
Source: F1000Res. 2026 Jul 14;13:840. Originally published 2024 Jul 26. [Version 2] doi: 10.12688/f1000research.146980.2 (PMC13653104; doi:10.12688/f1000research.146980.2)
Supplement: Peer review history

# Reviewer report: Reviewer response for version 2

**DOI:** 10.5256/f1000research.196845.r527959

**Published:** 2026-10-10

**Authors:** Chockalingam Nageswari

**Affiliations:** 1 Galgotias University, Greater Noida, Uttar Pradesh, India

1. The study protocol is generally well structured and addresses a clinically relevant research question. However, some methodological details require further clarification (what about the control group? are they not doing any exercises?) particularly regarding the standard-care protocol and monitoring of antihypertensive medication changes.

 2. The rationale for the selection and dosage/progression of the Mat Pilates intervention could be strengthened with supporting evidence especially the references for Pilates reducing the blood pressure are only mentioned in reference 27. Other than that, no other references for mat Pilates reducing the blood pressure. Try to add more references supporting the above point.

 3. About the exercise prescription the duration sets and repetition is not mentioned.

Is the study design appropriate for the research question?

Yes

Is the rationale for, and objectives of, the study clearly described?

Yes

Are sufficient details of the methods provided to allow replication by others?

Yes

Are the datasets clearly presented in a useable and accessible format?

Yes

Reviewer Expertise:

Physiotherapy in Obstetrics and Gynaecology, women's health, Biomechanics

I confirm that I have read this submission and believe that I have an appropriate level of expertise to confirm that it is of an acceptable scientific standard.

# Reviewer report: Reviewer response for version 1

**DOI:** 10.5256/f1000research.161112.r317117

**Published:** 2024-09-09

**Authors:** Susana Lopes

**Affiliations:** 1 School of Health Sciences and Institute of Biomedicine - iBiMED, University of Aveiro, Aveiro, Portugal; 2 ESTeSC Coimbra Health School, Polytechnic of Coimbra, Coimbra, Portugal; 3 Saúde Positiva, Clínica de Saúde Física e Mental, Aveiro, Portugal

This study protocol The aims to investigate the effectiveness of Mat Pilates in reducing blood pressure and improving quality of life in individuals with hypertension. A randomized controlled trial will compare the effects of Mat Pilates to standard care over 12 weeks. The primary outcome measures include resting and central blood pressure, health-related quality of life, and echocardiographic parameters.

 This is a well written and easy to read manuscript. 

 it is interesting, however, some issues could be addressed.

 Specific comments

 Abstract:

 Objective . Where we read "this RCT aimed to", should read "this RCT aims to "

 A two-arm, single-blinded, block randomized controlled trial will be recruited with120 participants into control and experimental groups (1:1 ratio). - Is confusing. Rephrase

 Introduction:

 Epidemiological data would benefit with more up to date references.

 Authors state a scarcity of studies studying the effects of pilates on blood pressure, but many studies have been published recently including systematic reviews and meta-analysis .

 Objectives - secondary outcomes

 Why did the authors choose "Two-dimensional (2D) echocardiographic parameters included the left ventricular ejection fraction (LVEF) and end-diastolic volume" as a secondary outcome for this population? Do you expect to see structural changes in the heart and ejection fraction in a 12-week mat pilates program?

 Eligibility criteria

 Why is systolic blood pressure >160 a exclusion criteria, since systolic blood pressure < 180 mm hg are usually eligible for exercise?

 Must there not be an interval before starting exercise while medication needs to be implemented. I mean, medication should be unchanged at least 1 month before starting exercise to avoid any change in blood pressure values due to medication

 why was a 2mmHg difference used to estimate sample size?

 I have some questions regarding the interventions. Will the exercise group not be taking any anti-hypertensive medication? Why is it just referred for the control group? This must be clarified.

 How to you intend to control for anti-hypertension medication intake? and to other exercise regimes in both groups?

Is the study design appropriate for the research question?

Yes

Is the rationale for, and objectives of, the study clearly described?

Yes

Are sufficient details of the methods provided to allow replication by others?

Partly

Are the datasets clearly presented in a useable and accessible format?

Not applicable

Reviewer Expertise:

Pilates, hypertension, cardiac rehabilitation

I confirm that I have read this submission and believe that I have an appropriate level of expertise to confirm that it is of an acceptable scientific standard, however I have significant reservations, as outlined above.

# Response

**Published:** 2026-02-19

**Authors:** Shivashankar KN

**Affiliations:** Medicine, Manipal Academy of Higher Education, Manipal, Karnataka, India

Specific comments

 Abstract:

 Objective: Where we read "this RCT aimed to", should read "this RCT aims to "

**Response: **Thank you for the correction. Phrase has been corrected.

 A two-arm, single-blinded, block randomized controlled trial will be recruited with120 participants into control and experimental groups (1:1 ratio). - Is confusing. Rephrase

**Response: **The statement has been rephrased. A two-arm, single-blinded, block randomized controlled trial will be recruited with a total of 120 participants with 60 participants each in the control and experimental groups.

 Introduction:

 Epidemiological data would benefit with more up to date references.

**Response: **Thank you for the valuable inputs. The data has been revised.

 Authors state a scarcity of studies studying the effects of pilates on blood pressure, but many studies have been published recently including systematic reviews and meta-analysis.

**Response: **The scarcity of studies reported are in reference to central blood pressure parameters when the protocol was designed in 2018. Many of the studies were later published which has been revised.

 Objectives - secondary outcomes

 Why did the authors choose "Two-dimensional (2D) echocardiographic parameters included the left ventricular ejection fraction (LVEF) and end-diastolic volume" as a secondary outcome for this population? Do you expect to see structural changes in the heart and ejection fraction in a 12-week mat pilates program?

**Response: ** As per the null and alternate hypothesis, we intend to explore and see if there would be any structural or functional changes of the myocardial systolic or diastolic function by measuring left ventricular end diastolic volume and ejection fraction through exercise, hence we have chosen 2D echocardiographic parameters as secondary outcomes. Existing literature suggests cardiac remodelling (structural changes) can begin and range between 3 months (12 weeks) up to 6 months (24 weeks) with regular exercise.

 For reference: 1) Garza MA, Wason EA, Zhang JQ. Cardiac remodeling and physical training post myocardial infarction. World J Cardiol. 2015 Feb 26;7(2):52-64. doi: 10.4330/wjc.v7.i2.52.

 2) Haykowsky M, Scott J, Esch B, Schopflocher D, Myers J, Paterson I, Warburton D, Jones L, Clark AM. A meta-analysis of the effects of exercise training on left ventricular remodeling following myocardial infarction: start early and go longer for greatest exercise benefits on remodeling. Trials. 2011 Apr 4;12:92. doi: 10.1186/1745-6215-12-92.

 Eligibility criteria

 Why is systolic blood pressure >160 as exclusion criteria, since systolic blood pressure <180 mm hg are usually eligible for exercise?

**Response: **In reference to the cut off values of 160 mm Hg, data from Indian studies report smaller diameter vessels (especially coronary artery vasculature) which are at a higher risk of early occlusion and earlier risk of CVD events (Stroke, Myocardial infarction).

 Hereby providing the reference studies for the selected exclusion criteria as 160 mmHg of systolic pressure.

1. Modi, Ranjan1; Modi, Sunil2. Coronary artery morphometrics in Indians – An optical coherence tomography perspective. Journal of Current Cardiology 2(2):p 105-108, May–Aug 2024. | DOI: 10.4103/JCC.JCC_11_23
2. Raut BK, Patil VN, Cherian G. Coronary artery dimensions in normal Indians. Indian Heart J. 2017 Jul-Aug;69(4):512-514. doi: 10.1016/j.ihj.2017.01.009.

In additiona, as suggested by the scientific review committees based on the above literature, 160 mm Hg was considered as the cut off value for systolic blood pressure.

 Must there not be an interval before starting exercise while medication needs to be implemented. I mean, medication should be unchanged at least 1 month before starting exercise to avoid any change in blood pressure values due to medication.

**Response:**

 Standard protocols for antihypertensive medications remain unchanged prior to the trial period for 4 weeks (1 month). However, various individual participant characteristics/variables (non- modifiable, e. g hereditary predisposition/familial hypertension, environmental and lifestyle related factors – humidity, temperature, hydration) additionally would need to be considered that may cause any variation in the blood pressure regulation during other times of the day.

 Why was a 2mmHg difference used to estimate sample size?

**Response:**

 The 2mm of Hg MCID was calculated from the pilot study (consisting of 10 participants) results conducted before the clinical trial in the Indian population on anti-hypertensive drug therapy.

 I have some questions regarding the interventions. Will the exercise group not be taking any anti-hypertensive medication? Why is it just referred for the control group? This must be clarified.

**Response:**

 Both the exercise and control groups will be receiving the standard medical care of antihypertensive medications during the trial. The medications are not exclusive to the control group alone. This has been revised.

 How to you intend to control for anti-hypertension medication intake? and to other exercise regimes in both groups?

**Response: **The antihypertensive medications will be prescribed by medical physicians of the participants. Any variation in the dosage of antihypertensive medications will be monitored and recorded during the study period. However, various individual participant characteristics/variables (non- modifiable, e. g hereditary predisposition/familial hypertension) additionally would need to be considered that may cause any variation in the dosage. These variables and the altered dosage of medications can be later analysed as covariates using ANCOVA.

# Reviewer report: Reviewer response for version 1

**DOI:** 10.5256/f1000research.161112.r308430

**Published:** 2024-08-21

**Authors:** Shakeel Ahmed

**Affiliations:** 1 Department of Physical Therapy, University of Florida, Gainesville, FL, USA

**Abstract:**

 The objective sentence is incomplete. Insert "is" after trial in the sentence.

**Methods:** rephrase to "A two-arm, single-blinded, block randomized controlled trial will recruit 120 participants"

**Article summary:** Strength and Limitations:

 1. 1 ^st^ and 2 ^nd^ points contradict your hypothesis stated in the abstract.

**Procedure:** Elaborate on what constitutes standard care for control group? What about physical activity? can the participants in the control group exercise at home or a gym ? is this controlled?

Is the study design appropriate for the research question?

Yes

Is the rationale for, and objectives of, the study clearly described?

Yes

Are sufficient details of the methods provided to allow replication by others?

Partly

Are the datasets clearly presented in a useable and accessible format?

Not applicable

Reviewer Expertise:

Pulmonary rehabilitation, Respiratory neuroplasticity, Cardiovascular Rehabilitation

I confirm that I have read this submission and believe that I have an appropriate level of expertise to confirm that it is of an acceptable scientific standard.
